# The Harveian Society

**Published:** 1897-03-27

**Authors:** 


### Medical Progress and Hospital Clinics.

[*The Editor will be glad to receive offers of co-operation and contributions from members of the profession. All letters should be addressed to* The Editor, at the Office, 28 & 29, Southampton Street, Strand, London, W.C.]

### THE HARVEIAN SOCIETY.

At the last meeting of the Harveian Society, held on March 18th, a paper was read by Dr. Robert Boxall on "Some Common Troubles following Childbirth." The greater portion of the paper, however, was devoted to a consideration of the question of puerperal fever, and he showed by tabulated statistics compiled from the Registrar-General's reports that it was more prevalent in private practice than was generally supposed. The tables shown were practically the same as those used by Dr. Boxall to illustrate his book on "Antiseptics in Midwifery," published in 1893, but were brought up to date; and by them he showed that puerperal fever had not diminished in the last 50 years, but had rather increased, especially in the provinces; that the death-rate from accidents of childbirth had diminished, especially in London; and that the total death-rate of childbirth in England and Wales remained practically unaltered for the last 50 years. In lying-in hospitals, however, the death-rate, which had been appalling, had during the same period markedly diminished, and puerperal fever had been practically abolished since the introduction of antiseptic methods. Several tables dealing with the statistics of the General Lying-in Hospital were also shown.

In the discussion which followed, interest centred especially on a table which had been exhibited to show how after the great fall in the mortality from septicæmia which had followed the use of perchloride of mercury at the General Lying-in Hospital, the death-rate had gone up again when for a time Salufer had been substituted for the perchloride of mercury for internal douching, although sublimate solution had still been used for the hands and for bathing the external parts. Dr. Cullingworth took upon himself the responsibility for the use of this substance, and said he had been led to it partly by the very favourable reports which he had received as to its chemical activity and as to the success which had attended its use in general surgery, and partly by the fact that they had had some very serious cases of mercurial poisoning, not fatal, but cases giving rise to very great anxiety, and it had seemed to him at that time very desirable to obtain some less poisonous material for internal use.

In regard to the choice of an antiseptic, Dr. Boxall thought that although they had had less success while using carbolic acid than when perchloride of mercury was employed, some of their greater success with the latter material might be due to greater general experience in the use of antiseptics, and greater saturation of all concerned with the antiseptic idea; and he was not prepared to say that if they were now to revert to carbolic acid they might not obtain as great a success as they now obtained with perchloride. At the same time they had now learned how to use the perchloride so as to avoid the risk of poisoning; the great thing being to administer the douche in such a manner that the antiseptic fluid should not be allowed to remain in the passages. The best position for the patient was lying on her back, and some pressure should be exercised upon the uterus, for although the douche might only be applied to the vagina, it was certain that the fluid might enter the uterus unless this was controlled. This was especially the case when the douche was administered with the patient lying on the side. As to the sort of tube used, glass or celluloid made a very good tube, but he generally employed a piece of soft india-rubber tubing or a double channelled glass tube.

As to the necessity for the routine use of the douche, it must be remembered that the conditions in hospitals, and especially in teaching hospitals, were somewhat different from those of private practice. In hospitals, where students or midwives were being trained, it was impossible to make distinctions. One general rule must be laid down and followed in all cases. But in private practice, he did not advise the general routine use of the douche by the nurse. He, however, always used a douche of 1 in 2,000 of perchloride of mercury during and just after a labour, and these douches he administered himself. He did not, however, in ordinary cases apply the douche to the interior of the uterus either in hospital or in private practice. The cleansing of the hands and of the external genitals by aid of perchloride solution was in all cases of the utmost importance.

